# Polyamine regulates tolerance to water stress in leaves of white clover associated with antioxidant defense and dehydrin genes via involvement in calcium messenger system and hydrogen peroxide signaling

**DOI:** 10.3389/fphys.2015.00280

**Published:** 2015-10-12

**Authors:** Zhou Li, Yan Zhang, Dandan Peng, Xiaojuan Wang, Yan Peng, Xiaoshuang He, Xinquan Zhang, Xiao Ma, Linkai Huang, Yanhong Yan

**Affiliations:** Department of Grassland Science, College of Animal Science and Technology, Sichuan Agricultural UniversityChengdu, China

**Keywords:** antioxidant, dehydrin, gene expression, oxidative damage, polyamine, regulation, white clover (*Trifolium repens*)

## Abstract

Endogenous polyamine (PA) may play a critical role in tolerance to water stress in plants acting as a signaling molecule activator. Water stress caused increases in endogenous PA content in leaves, including putrescine (Put), spermidine (Spd), and spermine (Spm). Exogenous application of Spd could induce the instantaneous H_2_O_2_ burst and accumulation of cytosolic free Ca^2+^, and activate NADPH oxidase and *CDPK* gene expression in cells. To a great extent, PA biosynthetic inhibitor reduced the water stress-induced H_2_O_2_ accumulation, free cytosolic Ca^2+^ release, antioxidant enzyme activities and genes expression leading to aggravate water stress-induced oxidative damage, while these suppressing effects were alleviated by the addition of exogenous Spd, indicating PA was involved in water stress-induced H_2_O_2_ and cytosolic free Ca^2+^ production as well as stress tolerance. Dehydrin genes (*Y*_2_*SK, Y*_2_*K*, and *SK*_2_) were showed to be highly responsive to exogenous Spd. PA-induced antioxidant defense and dehydrin genes expression could be blocked by the scavenger of H_2_O_2_ and the inhibitors of H_2_O_2_ generation or Ca^2+^ channels blockers, a calmodulin antagonist, as well as the inhibitor of CDPK. These findings suggested that PA regulated tolerance to water stress in white clover associated with antioxidant defenses and dehydrins via involvement in the calcium messenger system and H_2_O_2_ signaling pathways. PA-induced H_2_O_2_ production required Ca^2+^ release, while PA-induced Ca^2+^ release was also essential for H_2_O_2_ production, suggesting an interaction between PA-induced H_2_O_2_ and Ca^2+^ signaling.

## Introduction

Water deficit leads to decreases in crop yields which are associated with changes of various physiological and molecular factors. Recently, increasing evidence indicates that polyamine (PA) is closely involved in growth and development (Krasuska et al., 2014; Pottosin et al., 2014), as well as stress tolerance in plants (Yiu et al., 2009; Do et al., 2014). Maintenance of PA levels and metabolism, whether through exogenously applied putrescine (Put), spermidine (Spd) and spermine (Spd), or transgenic approaches with PA biosynthesis genes, has been found to promote plant stress tolerance due to their roles in protecting membranes, maintaining osmotic adjustment, and promoting tolerance-related gene expression and protein levels (Tang and Newton, 2005; Shi et al., 2013; Li et al., 2014a). It has been reported that PA synthesis and oxidation could improve H_2_O_2_-induced antioxidant protection in *Medicago sativa* (Guo et al., 2014). Diamine oxidase (CuAO) catalyzed the degradation of Put to produce H_2_O_2_, thereby elevating the Ca^2+^ level in guard cells of *Vicia faba* (An et al., 2008). All of the above suggest that PA plays an important role in signaling pathways and may activate multiple signal factors to cope with abiotic stresses.

The production of reactive oxygen species (ROS), such as superoxide and H_2_O_2_, is regarded as the most common response in plants under abiotic stress. High levels of ROS cause protein and lipid peroxidation, degradation of chlorophyll, and programmed cell death (Imlay and Linn, 1988; Yan et al., 2011; Wang et al., 2012), while low levels of ROS produced during early phases of stress response have a regulatory role acting as intermediate signaling molecules in plant cells (Bolwell et al., 1998; Zhang et al., 2006). Application of abscisic acid (ABA), brassinosteroid (BR), and salicylic acid (SA) all resulted in a temporary accumulation of H_2_O_2_ followed by a gain in stress tolerance in different plant species (Agarwal et al., 2005; Lu et al., 2009; Xia et al., 2009). PA has also been implicated in the regulation of H_2_O_2_ generation in plants, since H_2_O_2_ is generally one of by-products from Put oxidation catalyzed by CuAO (An et al., 2008). It has been shown that PA-derived H_2_O_2_ participated in stress-induced cell wall stiffening and maturation (Angelini et al., 2010). Spm oxidase acted as a mediator of ROS production in HIV-induced neuronal toxicity (Capone et al., 2013). H_2_O_2_ involvement in PA-induced cell death has been demonstrated in tobacco (*Nicotiana tabacum*) (Iannone et al., 2013). In spite of these studies, it's still unclear whether H_2_O_2_ signaling is involved in PA-induced tolerance to water stress and whether PA could induce other H_2_O_2_ generation pathways in plants under water stress.

ROS-related stress signaling may involve the calcium messenger system, which plays a critical role in coupling a wide range of extracellular stimuli with intracellular responses in plant cells. Calcium-signal-encoding elements such as Ca^2+^/H^+^ antiporters or Ca^2+^-ATPases regulate Ca^2+^ transport leading to the change of cytosolic Ca^2+^ concentrations (Shao et al., 2008). Ca^2+^ binds to different calcium sensors such as calmodulin (CaM) and Ca^2+^–dependent protein kinase (CDPK) to pass calcium signals as a result of transient cytosolic Ca^2+^ or oscillating Ca^2+^ levels (Sanders et al., 2002). The raising of cytosolic Ca^2+^ levels in guard cells can trigger stomatal closure, which demonstrates the importance of Ca^2+^ signaling for drought tolerance (Fu and Lu, 2007). ROS-induced Ca^2+^ influx across plasma membranes of *Araidopsis* was important for the growth of root hairs (Demidchik et al., 2007), and cytosolic Ca^2+^ could activate NOX to regulate plant adaptive responses (Pottosin et al., 2014). These studies indicate that there may be positive feedback between H_2_O_2_ and Ca^2+^ signaling in plants. PA has been found to induce stomatal closure by inducing Ca^2+^ release in *Arabidopsis* (Yamaguchi et al., 2007). Exogenous Ca^2+^ application elevated endogenous PA content, alleviating hypoxia damage in cucumber seedlings (*Cucumis sativus*) (Jiao et al., 2007). However, the cross-talk among PA, H_2_O_2_, and Ca^2+^ for water stress responses is not well-documented.

The PA-induced tolerance to water stress could be associated with the interaction between H_2_O_2_ and Ca^2+^ for the regulation of antioxidant systems. The objective of this study was to elucidate whether PA-induced stress tolerance involves the calcium messenger system and H_2_O_2_ signaling, the roles of PA signal transduction in antioxidant defense and dehydrins, and the interaction between H_2_O_2_ and Ca^2+^ induced by PA in plant cells.

## Materials and methods

### Plant materials

The white clover cultivar “Ladino” was used as plant material. All seeds were immersed in 0.1% mercuric chloride for 4 min and rinsed three times with distilled water. 0.1 g sterilized seeds were sown in trays (24 cm length, 20 cm width, and 15 cm deep) filled with sterilized quartz sand and distilled water, and then put in a controlled growth chamber (12 h photoperiod, 75% relative humidity, 23/19°C day/night temperature, and 500 μmol·m^−2^s^−1^ photosynthetically active radiation). After 7 days of germination in distilled water solution, the seedlings of white clover grew in full-strength Hoagland's solution (Hoagland and Arnon, 1950) for another 23 days (replacing the solution every other day). The second leaves were collected for all investigations.

### Treatments with chemicals

The detached leaves were pre-treated with distilled H_2_O for 1 h to eliminate wound stress and then placed in 50 ml centrifuge tubes containing distilled H_2_O, spermidine (Spd) or polyethyleneglycol (PEG) 6000 solution to induce water stress for various time periods under the same growth chamber conditions as mentioned above. In order to investigate the effects of different inhibitors or scavengers, the detached leaves were incubated in 20 μM Spd or 15% PEG solution separately containing 100 μM dicyclohexylamine (DCHA), an inhibitor of polyamine biosynthesis (Meskaoui and Trembaly, 2009); 5 mM dimethylthiourea (DMTU), a H_2_O_2_ scavenger (Lu et al., 2009); 100 μM Diphenyleneiodonium (DPI), an inhibitor of NADPH oxidase (Jiang and Zhang, 2002); 5 mM salicyhydroxamic (SHAM), an inhibitor of cell wall-localized peroxidase (Kiba et al., 1997); 100 μM quinacrine (QC), an inhibitor of amine oxidase (Kiba et al., 1997); 1 mM lanthanum (III) (LaCl_3_), a plasma membrane calcium channel blocker (Knight et al., 1992); 50 μM ruthenium red (RR), a mitochondrial, and endoplasmic reticulum calcium channel blocker (Knight et al., 1992); 150 μM N-(6-Aminohexyl)-5-chloro-1-naphthalenesulfonamide (W7), a calmodulin antagonist (Gonzalez et al., 2012) or 30 μM trifluoperazine (TFP), a inhibitor of Ca^2+^–dependent protein kinase (CDPK) (Lanteri et al., 2006). Detached leaves were incubated in distilled H_2_O as control under the same condition. All treatments were repeated at least 4–6 times (specific number of replicates and length of time about treatments with chemicals please see each figure legend).

### Endogenous polyamine determination by HPLC

Polyamines were extracted using the method of Duan et al. (2008). Detached leaves (0.25 g) were ground in 1 ml cold perchloric acid (5%, v: v) and the homogenate was incubated at 4°C for 1 h and then centrifuged for 30 min (12,000 rmp, 4°C). The supernatant was benzoylated as follows. Two milliliters NaOH (2 M) and 10 μl benzoyl chlorides were added into 500 μl supernatant and then they were incubated at 37°C for 30 min. In order to terminate the reaction, 2 ml saturated NaCl solution was added into the mixed solution. After that, 2 ml cold diethyl was used for extracting benzoyl polyamine. Finally, 1 ml of the diethyl ether phase was evaporated to dryness and re-dissolved in 1 ml methanol for determination of endogenous polyamines. High performance liquid chromatography (Agilent-1200, Agilent Technologies, USA) was used for analyzing endogenous PA content. 20 μl of benzoyl PA was injected into loop and then loaded onto a reverse-phase Tigerkin®C18 column (150 × 4.6 mm, 5 μm particle size). Column temperature was maintained at 25°C. Mobile phase was methanol-H_2_O (64: 36, v: v). PA peak was detected at a flow rate of 1 ml min^−1^ at 254 nm.

### Determination of water deficit and oxidative damage

The leaf relative water content (RWC) was measured as an indicator of water deficit by using the formula RWC (%) = [(FW-DW)/(TW-DW)] × 100 (FW, fresh weights; TW, turgid weights; and DW, dry weights). Treated leaves (0.1 g) in different chemical solutions were collected, gently blotted dry, and weighed for FW. After immersing these leaves in deionized water for 16 h at 4°C in the dark, TW was measured. Samples were then dried in an 80°C oven for more than 72 h for weighing DW (Barrs and Weatherley, 1962). For extraction of malondialdehyde (MDA), the leaves (0.1 g) were ground with 2 ml PBS (50 mM, pH 7.8) and then centrifuged for 30 min (12,000 rpm, 4°C). The 0.5 ml supernatant was added to 1 ml reaction solution (20% trichloroacetic acid, 0.5% thiobarbituric acid) and then heated for 15 min at 95°C. After cooling quickly, the mixture was centrifuged for 10 min at 8000 rpm. The absorbance of the supernatant was calculated by subtraction of OD_600_ from OD_532_ (Dhindsa et al., 1981). Electrolyte leakage (EL) was measured to evaluate plasma membrane stability. Leaves (0.1 g) were placed in centrifuge tubes containing 35 ml of distilled H_2_O for 24 h. The initial conductance (C_*i*_) was measured using a conductivity meter (YSI Model 32, Yellow Springs, OH). Tubes were then put in an autoclave for 20 min at 140°C and the maximal conductance (C_max_) was measured. Finally, EL was calculated according to the formula (%) = C_i_/C_max_ × 100 (Blum and Ebercon, 1981). Protein carbonyl content was analyzed by using a Protein Carbonyl Colorimetric Assay Kit (Cayman Chemical, USA) and the absorbency of reaction solution was determined on a microplate reader (Synergy HTX, Bio Tek, USA).

### Cytosolic free Ca^2+^ and H_2_O_2_ detection by CLSM

H_2_O_2_ and cytosolic free Ca^2+^ were detected by using a H_2_O_2_-sensitive fluorescent probe H_2_DCFDA (Sigma, USA) and free cytosolic Ca^2+^-specific fluorescent Fluo-3 AM probe (Beyotime, China). In order to eliminate wound stress, the detached leaves were pre-treated with distilled H_2_O for 1 h and then incubated in distilled H_2_O to act as a control or 20 μM Spd or 15% PEG for different times or chemical treatments (specifically described in each figure legend) in growth chamber conditions as mentioned above, followed by loading with 25 μM H_2_DCFDA or 2.5 μM Fluo-3 AM for 30 min in the dark. After that, detached leaves were rinsed three times in Tris-KCl buffer (pH 7.2) until all fluorescent probes were removed from the foliar surface. A confocal laser scanning microscope (Nikon A1, Nikon, Japan) was used for visualizing all images (excitation 488, emission 525). For measurement of H_2_O_2_ content, 0.2 g fresh leaves were ground with 4 ml of 5% TCA and 0.15 g activated charcoal and then centrifuged at 10000 rpm for 20 min at 4°C. The 3 ml supernatant was adjusted to pH 8.4 with 17 M ammonia solution and then filtered. One milliliter of colorimetric reagent (10 mg of 4-aminoantipyrine, 10 mg of phenol, and 5 mg of peroxidase) was added into the filtrate with and without catalase. The reaction solution was incubated for 10 min at 30°C. Absorbance was determined at 505 nm on a microplate reader (Zhou et al., 2006).

### Determination of antioxidant enzyme and NADPH oxidase activity

For antioxidant enzyme activity assays, the supernatant was collected according to the procedure for MDA extraction as mentioned above. For determination of superoxide dismutase (SOD) activity, 1.5 ml reaction solution (50 mM PBS containing 195 mM methionine, 60 μM riboflavin, and 1.125 mM NBT) was added into 0.05 ml enzyme extract and the change of absorbance was recorded at 560 nm (Giannopolitis and Ries, 1977). The activities of guaiacol peroxidase (GPOX) and catalase (CAT) were measured based on the methods of Chance and Maehly (1955). Briefly, 0.05 ml enzyme extract was mixed with 1.5 ml reaction solution (0.05 ml of 0.75% H_2_O_2_, 0.5 ml of 0.25% guaiacol solution, and 0.995 ml of 100 mM PBS, pH 5.0 for the assay of GPOX or 0.5 ml of 45 mM H_2_O_2_ and 1 ml of 50 mM PBS, pH 7.0 for the assay of CAT). The changes in absorbance were monitored at 460 or 240 nm every 10 s for 1 min for GPOX and CAT, respectively. For measurement of ascorbate peroxidase (APX) activity, 0.05 ml of enzyme extract was added into 1.5 ml of reaction solution containing 10 mM ascorbic acid, 0.003 mM EDTA, 5 mM H_2_O_2_ and 100 mM PBS (pH 5.8) and the change of absorbance was recorded every 10 s for 1 min at 290 nm (Nakano and Asada, 1981). The method of two-phase aqueous polymer partition system was used for obtaining of leaf plasma membrane (Larsson et al., 1987). One milliliter reaction solution contains 20 μl NADPH (50 μM), 100 μl XTT (0.5 mM), 780 μl Tris-HCl (pH 7.5) and 20 μg plasma membrane protein. The blank was added with 20 μl SOD (100 U mg^−1^). After 5 min at 25°C, the reaction solution was used for spectrophotometric analysis at A470. ΔA470 represents the difference in XTT (3′-[l-[phenylamino-carbonyl]-3,4-tetrazolium]-bis (4-methox-6-naitro)) absorbance in the presence and absence of 100 units of SOD at 470 nm (Jiang and Zhang, 2002). Bradford's (1976) method was used for determining protein content.

### Total RNA extraction and qRT-PCR analysis

Transcript levels of antioxidant enzyme genes were performed using real-time quantitative polymerase chain reaction (qRT-PCR). For total RNA extraction, the detached leaves of white clover were extracted by using RNeasy Mini Kit (Qiagen, Germany) according to the manufacturer's instructions. A cDNA Synthesis Kit (Fermentas, Lithuania) was used for reverse-transcribing RNA to cDNA. Primer sequences for the genes *SOD, GPOX, CAT*, and *APX, CDPK, Y*_2_*SK, Y*_2_*K, SK*_2_ (Vaseva et al., 2011) and β*-Actin* (internal control) are shown in Table 1. Gene expression levels were determined using an iCycler iQ qRT-PCR detection system with SYBR Green Supermix (Bio-Rad). The conditions of the PCR protocol for all genes were as follows: 5 min at 95°C and 40 repeats of denaturation at 95°C for 15 s, annealing at 58°C (*SOD, CAT, GPOX*, and *SK*_2_ gene), or 60°C (*Y*_2_*SK*, and *Y*_2_*K*) or 63°C (*APX* and *CDPK* gene) for 45 s, following by heating the amplicon from 60 to 95°C to obtain the melting curve. At the end of PCR cycle, the transcript level of all genes was calculated according to the formula 2^−ΔΔ*Ct*^ described by Xia et al. (2009).

**Table 1:** **Primer sequences and their corresponding GeneBank accession numbers of the analyzed genes**.

**Target gene**	**Accession no**.	**Forward primer (5′–3′)**	**Reverse primer (5′–3′)**
*SOD*	FY461274	TCACCTTCCACTTTCAAACCTCTC	TGTTGGACCTTCGTCTTCTTGAGT
*CAT*	FY464988	GTCTTCTTTGTTCACGATGGGATG	GAAAGTGGGAGAAGAAGTCAAGGAT
*GPOX*	AJ011939	TCTAGGGCAACGGTTAATTCATTC	GGTACGGATTTTCCCATTTCTTG
*APX*	FY460674	GCAGCATCAGTTGGCAAGACC	GGCAAACCTGAGACTAAATACACGA
*CDPK*	FY465549.1	CAAACCGGCATCCAATGACTT	GCATTCGTTTCAAGGAGGCATT
Dehydrin, *Y_2_SK*	GU443965.1	GTGCGATGGAGATGCTGTTTG	CCTAATCCAACTTCAGGTTCAGC
DHN1, *Y_2_K*	JF748410.1	AGCCACGCAACAAGGTTCTAA	TTGAGGATACGGGATGGGTG
Dhn b, *SK_2_*	GU443960.1	TGGAACAGGAGTAACAACAGGTGGA	TGCCAGTTGAGAAAGTTGAGGTTGT
*β-Actin*	JF968419	TTACAATGAATTGCGTGTTG	AGAGGACAGCCTGAATGG

### Statistical analysis

The data was analyzed by using SAS 9.1 (SAS Institute, Cary, NC). Significant differences among treatments were determined using Fischer's least significance difference (LSD) at the 0.05 probability level.

## Results

### PA is involved in tolerance to water stress in leaves of white clover

**Figure 1:**
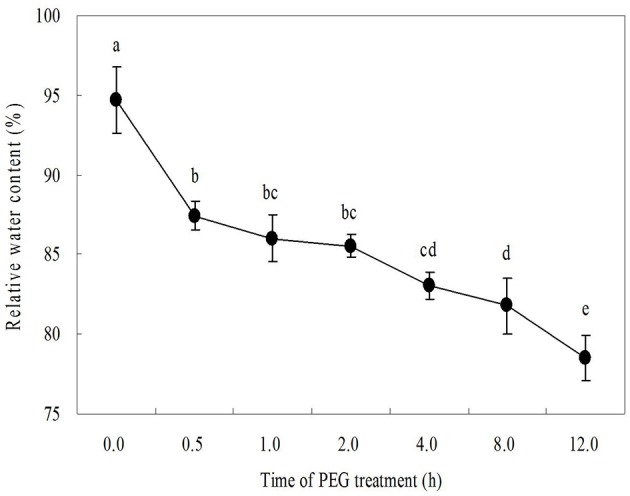
**Time course of PEG 6000-induced the change of leaf relative water content (%) as an indicator of water deficit**. The detached leaves were pre-treated with distilled water for 1 h to eliminate wound stress and then exposed to 15% PEG solution for 12 h. Means of six independent samples are presented. Bars represent standard errors. The same letter indicates no significant difference (LSD) at *P* < 0.05.

**Figure 2:**
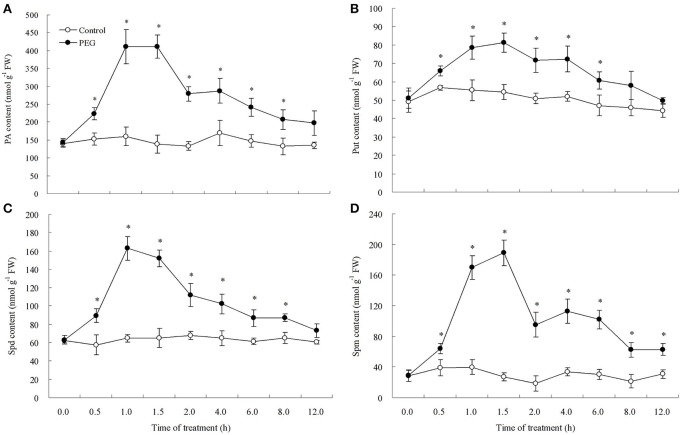
**Time course of PEG 6000-induced polyamine (PA) (A), putrescine (Put) (B), spermidine (Spd) (C), and spermine (Spm) accumulation (D) in leaves**. The detached leaves were pre-treated with distilled water for 1 h to eliminate wound stress and then exposed to distilled water (control) or 15% PEG solution for 12 h. Means of four independent samples are presented. Bars represent standard error. “*” indicate LSD values where significant differences were detected (*P* < 0.05) between two treatments at a given time.

**Figure 3:**
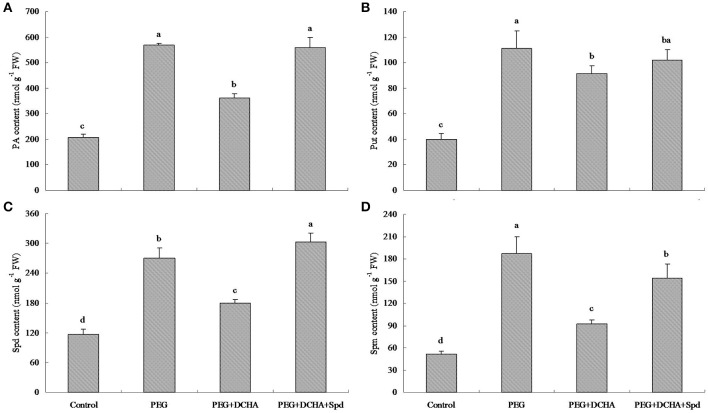
**Effects of spermidine (Spd) and inhibitor (DCHA) of aminopropyl transferase on PEG 6000-induced polyamine (PA) (A), putrescine (Put) (B), spermidine (Spd) (C), and spermine (Spm) accumulation (D) in leaves**. The detached leaves of white clover were pre-treated with distilled water for 1 h to eliminate wound stress and then treated for 1 h as follows: 1, distilled water (control); 2, 15% PEG; 3, 15% PEG + 100 μM dicyclohexylamine (DCHA); 4, 15% PEG + 100 μM DCHA + 20 μM Spd. Means of four independent samples are presented. Bars represent standard errors. The same letter above columns indicates no significant difference (LSD) at *P* < 0.05.

**Figure 4:**
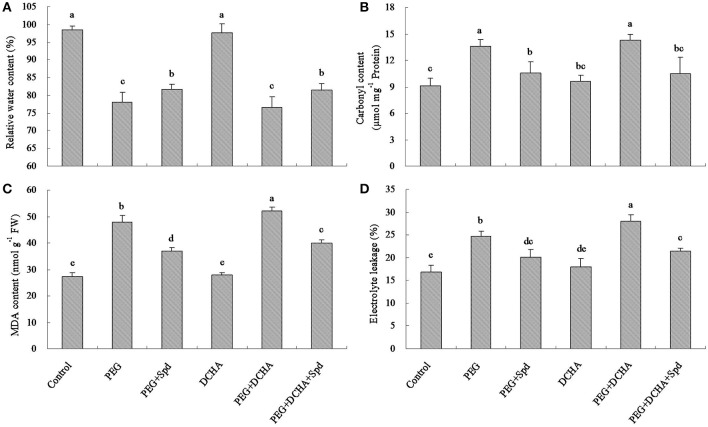
**Effects of spermidine (Spd) and inhibitor (DCHA) of aminopropyl transferase on PEG 6000-induced relative water content (A), carbonyl content (B), malondialdehyde content (C) and electrolyte leakage (D)**. The detached leaves of white clover were pre-treated with distilled water for 1 h to eliminate wound stress and then treated for 12 h as follows: 1, distilled water (control); 2, 15% PEG; 3, 15% PEG + 20 μM Spd; 4, 100 μM dicyclohexylamine (DCHA); 5, 15% PEG + 100 μM dicyclohexylamine (DCHA); 6, 15% PEG + 100 μM DCHA + 20 μM Spd. Means of six independent samples are presented. Bars represent standard errors. The same letter indicates no significant difference (LSD) at *P* < 0.05.

As shown in Figure 1, 15% PEG caused the decline in leaf relative water content (RWC). Only after 0.5 h, leaf RWC decreased to a significant lower degree. When treated in 15% PEG solution for 12 h, leaf RWC fell from almost 95% to only 78% (Figure 1). Water deficit induced changes in endogenous PA content in leaves of white clover (Figure 2). Endogenous PA, Put, Spd, and Spm content increased significantly at 0.5 h of water stress caused by 15% PEG and then reached a peak value at 1 h as compared with the control (treated with water). After 2 h of water stress, PA began to decline gradually in leaves (Figure 2). As shown in Figure 3, the inhibitor of aminopropyl transferase “DCHA” effectively inhibited the PEG-induced increase in endogenous PA, including Put, Spd, and Spm. However, exogenously applied Spd could reverse DCHA-induced decrease in PA content under water stress (Figure 3). In order to investigate whether PA was involved in tolerance to water stress in leaves, the effects of exogenous Spd and DCHA on leaf RWC, carbonyl content, MDA content, and EL level were tested in detached leaves under water stress (Figure 4). Exogenous Spd significantly improved leaf RWC under water stress. In addition, water stress caused oxidative damage in detached leaves, as reflected by increasing in protein oxidation (carbonyl content), lipid peroxidation level (MDA content), and membrane leakage (EL). These three indicators significantly declined with the application of exogenous Spd in response to water stress. However, DCHA caused further increase in PEG-induced carbonyl content, MDA content, and EL levels, but these negative effects of DCHA under water stress were alleviated by addition of 20 μM Spd.

### PA is involved in PEG-induced cytosolic free Ca^2+^ and H_2_O_2_ production

**Figure 5:**
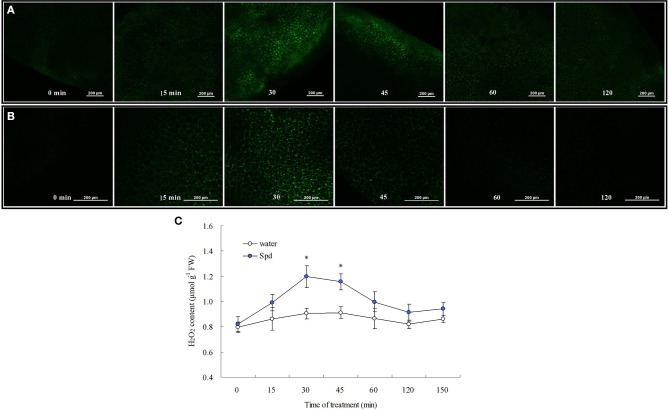
**Cytosolic free Ca^2+^ (A) and H_2_O_2_ production (B,C) as affected by spermidine (Spd) at different times**. The detached leaves were pre-treated with distilled water for 1 h to eliminate wound stress and then exposed to 20 μM Spd for various time, followed by incubation with Ca^2+^-sensitive fluorescent probe Fluo-3-AM or H_2_O_2_-sensitive fluorescent probe H_2_DCFDA for 30 min. Images are visualized using confocal laser scanning microscopy (CLSM). Means of four independent samples are presented. Bars represent standard error. “*” indicate LSD values where significant differences were detected (*P* < 0.05) between two treatments at a given time.

**Figure 6:**
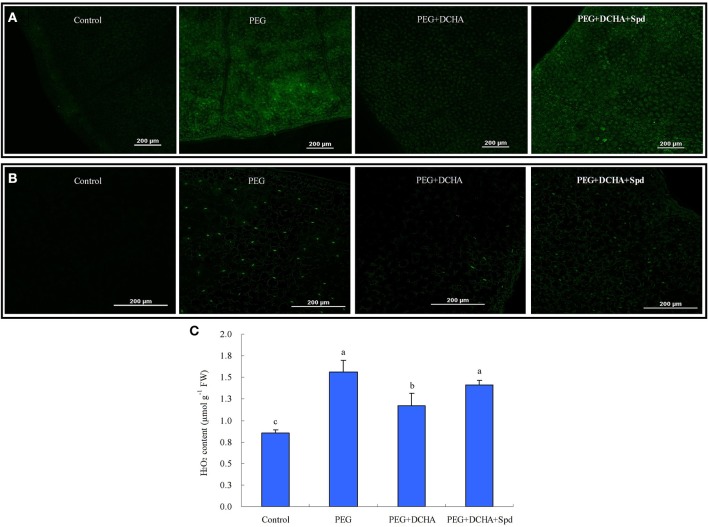
**Effects of polyamine (PA) on PEG-induced cytosolic free Ca^2+^ (A) and H_2_O_2_ production (B,C)**. The detached leaves of white clover were pre-treated with distilled water for 1 h to eliminate wound stress and then treated for 45 min as follows: 1, distilled water (control); 2, 15% PEG; 3, 15% PEG + 100 μM dicyclohexylamine (DCHA); 4, 15% PEG + 100 μM DCHA + 20 μM Spd and followed by incubation with Ca^2+^-sensitive fluorescent probe Fluo-3-AM or H_2_O_2_-sensitive fluorescent probe H_2_DCFDA for 30 min. Images are visualized using confocal laser scanning microscopy (CLSM). Means of four independent samples are presented. Bars represent standard errors. The same letter indicates no significant difference (LSD) at *P* < 0.05.

**Figure 7:**
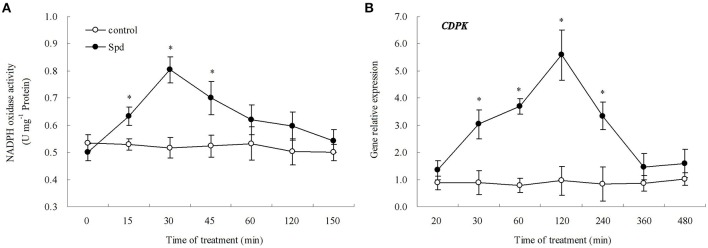
**Time course of spermidine (Spd)-induced NADPH oxidase activity (A), and *CDPK* gene relative expression (B) in leaves**. The detached leaves were pre-treated with distilled water for 1 h to eliminate wound stress and then exposed to distilled water (control) or 20 μm Spd solution for 480 min. Means of four independent samples are presented. Bars represent standard error. “*” indicate LSD values where significant differences were detected (*P* < 0.05) between two treatments at a given time.

A rapid change of cytosolic free Ca^2+^ was caused by exogenous Spd in detached leaves. The Fluo-3 AM fluorescent probe signal reached a maximum at 30 min, and remained high at 45 min. After that, cytosolic free Ca^2+^ subsided to the normal level almost as quickly as it increased (Figure 5A). After 15 min treatment in Spd solution, cytosolic H_2_O_2_ generation in detached leaves was also observed (Figure 5B). Cytosolic H_2_O_2_ content reached a maximum at 30 min and then declined, which suggested that Spd induced instantaneous H_2_O_2_ burst in cells (Figures 5B,C). In Figure 6, it's shown that the treatment with 15% PEG for 45 min resulted in a significant accumulation of cytosolic free Ca^2+^ and H_2_O_2_, whereas 100 μM DCHA effectively blocked the accumulation of Ca^2+^ and H_2_O_2_ induced by 15% PEG. The application of exogenous Spd markedly restored the increase of cytosolic free Ca^2+^ and H_2_O_2_ blocked by DCHA in detached leaves exposed to 15% PEG (Figure 6). Exogenous Spd affected the time-course changes of NADPH oxidase activity and *CDPK* gene expression (Figure 7).

### PA is involved in PEG-induced antioxidant enzyme activities and genes expression

**Figure 8:**
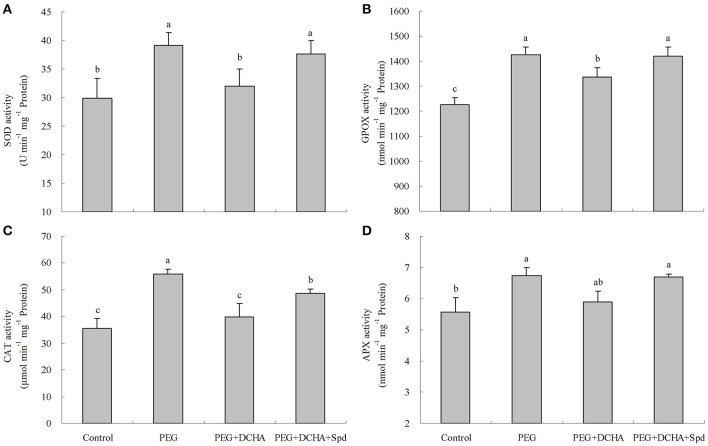
**Antioxidant activities as affected by polyamines (PA) under PEG-induced water stress**. SOD **(A)**, GPOX **(B)**, CAT **(C)**, and APX **(D)** The detached leaves of white clover were pre-treated with distilled water for 1 h to eliminate wound stress and then treated for 8 h as follows: 1, distilled water (control); 2, 15% PEG; 3, 15% PEG + 100 μM dicyclohexylamine (DCHA); 4, 15% PEG + 100 μM DCHA + 20 μM Spd. Means of six independent samples are presented. Bars represent standard errors. The same letter indicates no significant difference (LSD) at *P* < 0.05.

**Figure 9:**
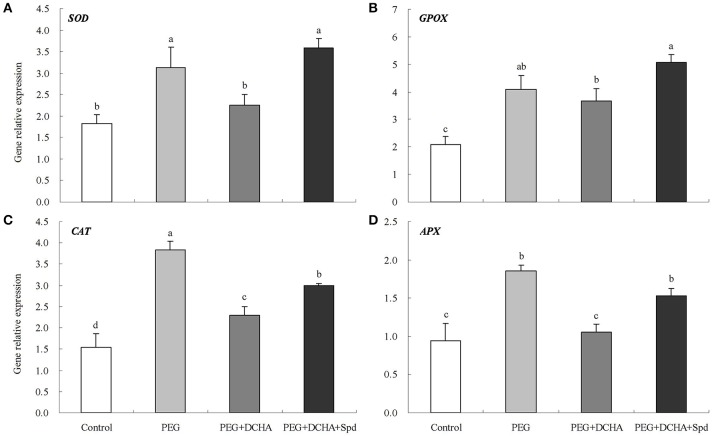
**Gene transcript levels encoding antioxidant enzymes as affected by polyamines (PA) under PEG-induced water stress**. *SOD* gene **(A)**, *GPOX* gene **(B)**, *CAT* gene **(C)**, and *APX* gene **(D)**. The detached leaves of white clover were pre-treated with distilled water for 1 h to eliminate wound stress and then treated for 2 h as follows: 1, distilled water (control); 2, 15% PEG; 3, 15% PEG + 100 μM dicyclohexylamine (DCHA); 4, 15% PEG + 100 μM DCHA + 20 μM Spd. Means of four independent samples are presented. Bars represent standard errors. The same letter indicates no significant difference (LSD) at *P* < 0.05.

Different PEG concentrations affected antioxidant enzyme activities (SOD, GPOX, CAT, and APX) in detached leaves as shown in Figure S1 and lower PEG concentrations (10 and 15%) significantly improved four enzyme activities. With increases in PEG concentration, antioxidant enzyme activities decreased gradually. Higher PEG concentrations (25%) failed to improve the four enzyme activities and even suppressed APX activity compared with the control (0% PEG) after 8 h of treatment (Figure S1D). Transcript levels of four genes encoding antioxidant enzymes were also up-regulated under 15% PEG (Figure S3). SOD, GPOX, CAT, and APX activities were enhanced by 31, 16, 57, and 21%, respectively after 15% PEG treatment for 8 h (Figure 8). To a certain extent, the PEG-induced effects on antioxidant enzyme activities were nullified when DCHA was added into 15% PEG solution, but these effects were reversed by the addition of Spd into 15% PEG and DCHA solution (Figure 8). PEG, DCHA, and Spd had similar effects on *SOD, GPOX, CAT*, and *APX* gene transcript levels (Figure 9).

### H_2_O_2_ and Ca^2+^ messenger systems are involved in Spd-induced antioxidant defense and dehydrin genes expression

**Figure 10:**
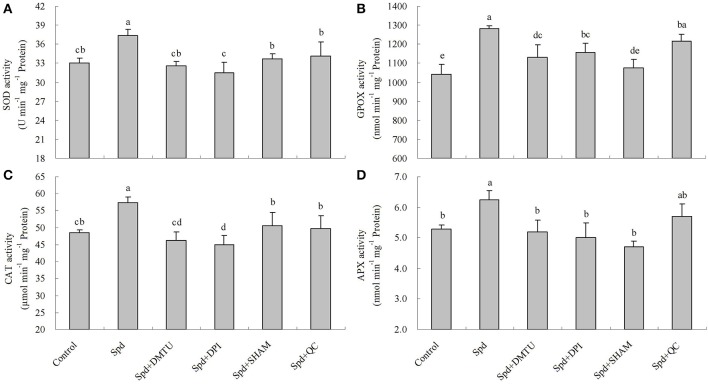
**Spd-induced SOD (A), GPOX (B), CAT (C), and APX (D) activities were dependent on hydrogen peroxide (H_2_O_2_) signaling**. The detached leaves of white clover were pre-treated with distilled water for 1 h to eliminate wound stress and then treated for 8 h as follows: 1, distilled water (control); 2, 20 μM Spd; 3, 20 μM Spd + 5 mM dimethylthiourea (DMTU); 4, 20 μM Spd + 100 μM Diphenyleneiodonium (DPI); 5, 20 μM Spd + 5 mM salicyhydroxamic (SHAM); 6, 20 μM Spd + 100 μM quinacrine (QC). Means of six independent samples are presented. Bars represent standard errors. The same letter indicates no significant difference (LSD) at *P* < 0.05.

**Figure 11:**
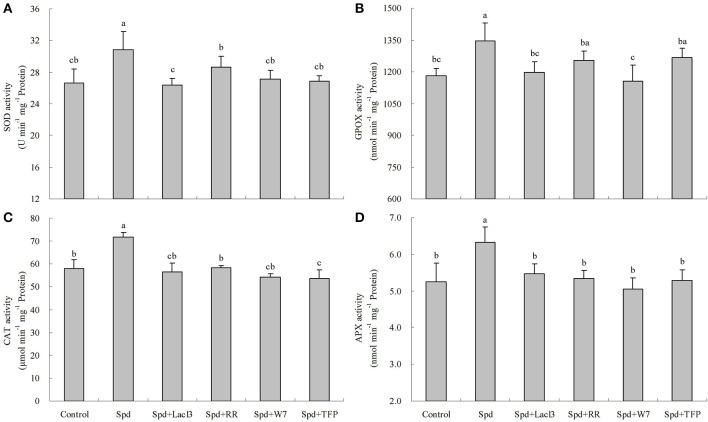
**Spd-induced SOD (A), GPOX (B), CAT (C), and APX (D) activities were dependent on calcium (Ca^2+^) messenger system**. The detached leaves of white clover were pre-treated with distilled water for 1 h to eliminate wound stress and then treated for 8 h as follows: 1, distilled water (control); 2, 20 μM Spd; 3, 20 μM Spd + 1 mM LaCl_3_; 4, 20 μM Spd + 50 μM ruthenium red (RR); 5, 20 μM Spd + 150 μM N-(6-Aminohexyl)-5-chloro-1- naphthalenesulfonamide (W7); 6, 20 μM Spd + 30 μM trifluoperazine (TFP). Means of six independent samples are presented. Bars represent standard errors. The same letter indicates no significant difference (LSD) at *P* < 0.05.

**Figure 12:**
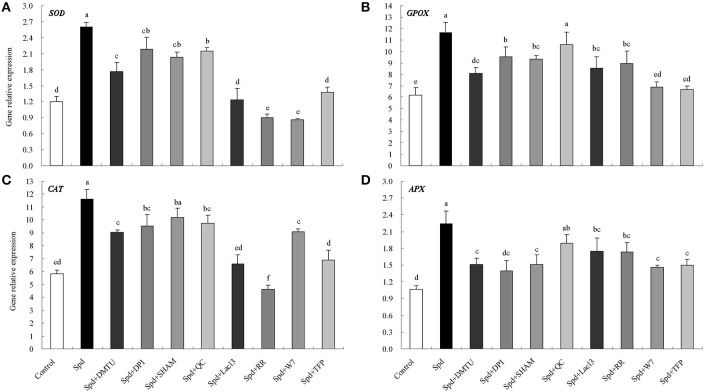
**Spd-induced *SOD* (A), *GPOX* (B), *CAT* (C), and *APX* (D) gene transcript levels were dependent on hydrogen peroxide (H_2_O_2_) signaling and calcium (Ca^2+^) messenger system**. The detached leaves of white clover were pre-treated with distilled water for 1 h to eliminate wound stress and then treated for 2 h as follows: 1, distilled water (control); 2, 20 μM Spd; 3, 20 μM Spd + 5 mM dimethylthiourea (DMTU); 4, 20 μM Spd + 100 μM Diphenyleneiodonium (DPI); 5, 20 μM Spd + 5 mM salicyhydroxamic (SHAM); 6, 20 μM Spd + 100 μM quinacrine (QC); 7, 20 μM Spd + 1 mM LaCl_3_; 8, 20 μM Spd + 50 μM ruthenium red (RR); 9, 20 μM Spd + 150 μM N-(6-Aminohexyl)-5-chloro-1-naphthalenesulfonamide (W7); 10, 20 μM Spd + 30 μM trifluoperazine (TFP). Means of four independent samples are presented. Bars represent standard errors. The same letter indicates no significant difference (LSD) at *P* < 0.05.

**Figure 13:**
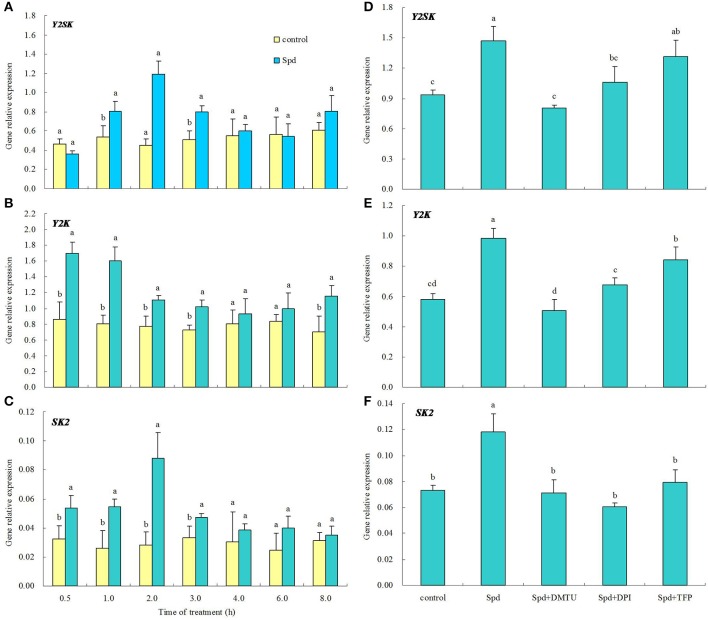
**Spd-induced dehydrins *Y*_2_*SK, Y*_2_*K* and *SK*_2_ genes relative expression were dependent on hydrogen peroxide (H_2_O_2_) and CDPK**. The detached leaves of white clover were pre-treated with distilled water for 1 h to eliminate wound stress and then treated for different times **(A–C)** or 2 h **(D–F)** as follows: 1, distilled water (control); 2, 20 μM Spd; 3, 20 μM Spd + 5 mM dimethylthiourea (DMTU); 4, 20 μM Spd + 100 μM Diphenyleneiodonium (DPI); 5, 20 μM Spd + 30 μM trifluoperazine (TFP). Means of four independent samples and standard errors are presented. The different letter above the columns between two treatments at a given time indicates significant difference at *P* < 0.05 **(A–C)**. The same letter above the columns indicates no significant difference at *P* < 0.05 **(D–F)**.

Figure S2 showed the effects of Spd concentrations on elevating the enzyme activities of SOD, GPOX, CAT, and APX. Three concentrations of Spd (15, 20, and 25 μM) significantly enhanced antioxidant enzyme activities after 8 h of treatment. Among these concentrations, 20 μM Spd exhibited the highest induction of activity on the four enzymes (Figure S2). Treatment with 20 μM Spd also led to significant up-regulation of gene transcript levels for *SOD, GPOX, CAT*, and *APX* within 8 h of treatment (Figure S3). To investigate whether H_2_O_2_ signaling and the calcium messenger system were involved in Spd-induced antioxidant enzyme activities and genes expression, a H_2_O_2_ scavenger, enzyme inhibitors, and calcium channel blockers were tested. Spd-induced enzyme activities and genes expression (*SOD, GPOX, CAT*, and *APX*) were suppressed to a certain extent when H_2_O_2_ scavenger (DMTU), NADPH oxidase inhibitor (DPI), cell wall-localized peroxidase inhibitor (SHAM), or amine oxidase inhibitor (QC) was added to the Spd solution (Figures 10, **12**). Moreover, DMTU and DPI showed the greatest effects on reducing Spd-induced antioxidant enzyme activities and genes expression, while the effect of QC was the least. Similarly, treatment with Ca^2+^ channel blockers (plasma membrane calcium channel blocker, LaCl_3_; inhibitor of mitochondrial and endoplasmic reticulum calcium channels, RR), calmodulin antagonist (W7), or an inhibitor of CDPK (TFP) significantly blocked the increases in antioxidant enzyme activities and gene transcript levels induced by Spd (Figures 11, 12). Detached leaves treated with 20 μM Spd led to significant changes in three dehydrin gene transcript levels relative to the control at different times (Figures 13A–C). However, DMTU, DPI, and TPF significantly reduced Spd-induced dehydrin genes expression including *Y*_2_*SK, Y*_2_*K*, and *SK*_2_ (Figures 13D–F).

### The interaction of Spd-induced H_2_O_2_ and Ca^2+^

**Figure 14:**
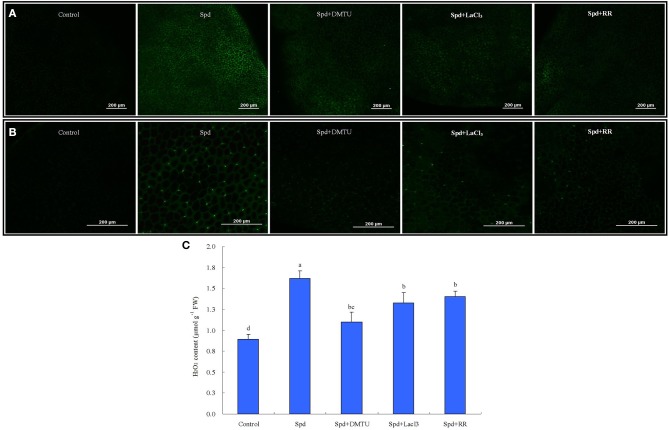
**Cytosolic free Ca^2+^ (A) and H_2_O_2_ accumulation (B–C) in response to spermidine (Spd), H_2_O_2_scavenger or calcium (Ca^2+^) channels blockers**. The detached leaves of white clover were pre-treated with distilled water for 1 h to eliminate wound stress and then treated for 30 min as follows: 1, distilled water (control); 2, 20 μM Spd; 3, 20 μM Spd + 5 mM dimethylthiourea (DMTU); 4, 20 μM Spd + 1 mM LaCl_3_; 5, 20 μM Spd + 50 μM ruthenium red (RR) and followed by incubation with Ca^2+^-sensitive fluorescent probe Fluo-3-AM or H_2_O_2_-sensitive fluorescent probe H_2_DCFDA for 30 min. Images are visualized using confocal laser scanning microscopy (CLSM). Means of four independent samples are presented. Bars represent standard errors. The same letter indicates no significant difference (LSD) at *P* < 0.05.

**Figure 15:**
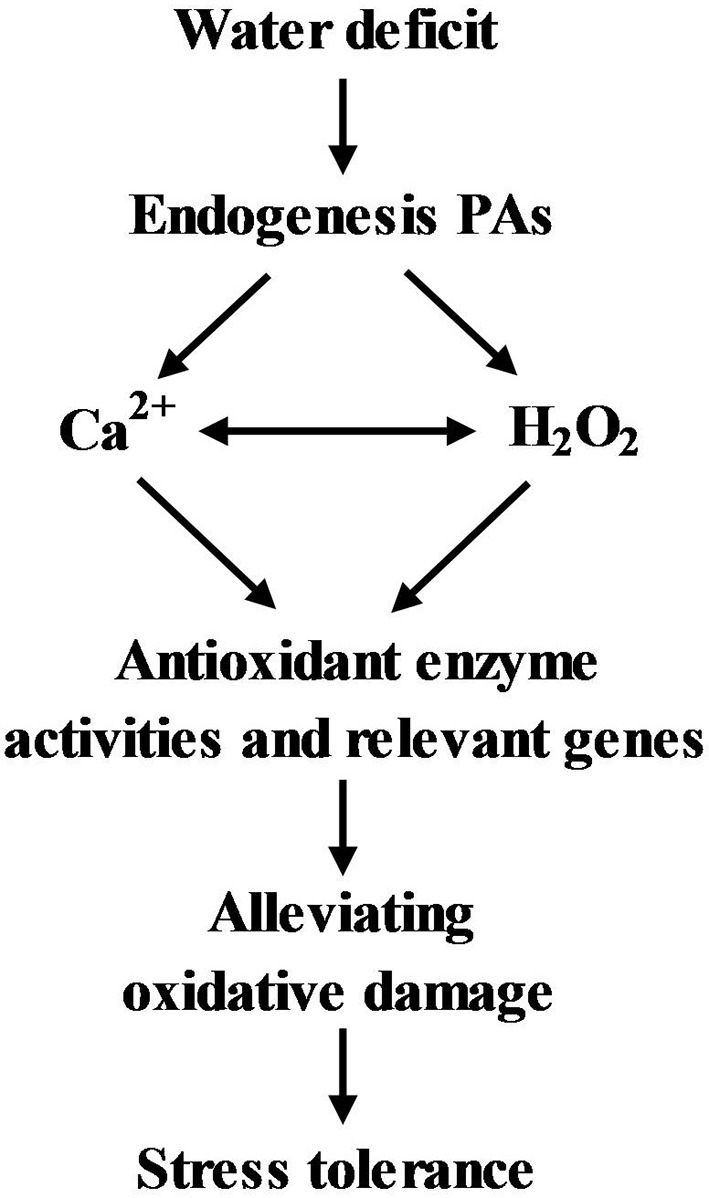
**A model for the polyamine (PA) regulation of Ca^2+^ and H_2_O_2_ signaling in control of antioxidant defense involved in tolerance to water stress in white clover**.

The interaction between Spd-induced H_2_O_2_ and Ca^2+^ was further investigated (Figure 14). Cytosolic free Ca^2+^ content increased to higher levels after treatment with 20 μM Spd for 30 min (Figure 14A). However, the change in free Ca^2+^ content was not detected in the control after the same length of time. The enhanced Ca^2+^ fluorescence was inhibited by calcium channel blockers LaCl_3_ and RR in detached leaves, and was also eliminated by H_2_O_2_ scavenger (DMTU), indicating that Spd-induced cytosolic free Ca^2+^ accumulation was dependent upon H_2_O_2_ signaling (Figure 14A). Similarly, a Spd-induced H_2_O_2_ increase in cells was significantly blocked by application of LaCl_3_, RR, and DMTU, which showed that Spd-induced H_2_O_2_ accumulation was involved in Ca^2+^ signaling (Figures 14B,C). Based on all above findings, we could deduce a model for PA regulation of Ca^2+^ and H_2_O_2_ signaling in control of antioxidant defense involves in tolerance to water stress in leaves of white clover (Figure 15).

## Discussion

### PA is involved in tolerance to water stress in white clover

It has been observed that abiotic stress can induce increases in endogenous PA content in various plant species, which is important for adaptation to drought stress (Kusano et al., 2008; Zhou and Yu, 2010; Hu et al., 2012). Elevated endogenous PA content through exogenously applied Spd or Spm led to enhancement in drought tolerance in *Arabidopsis thaliana* (Kusano et al., 2007), rice (*Oryza sativa*) (Farooq et al., 2009), and creeping bentgrass (*Agrostis stolonifera*) (Li et al., 2015). In the current study, our results showed that endogenous PA content increased significantly in detached clover leaves under water stress. However, DCHA, an inhibitor of PA biosynthesis, suppressed water stress-induced increases in endogenous PA levels, in agreement with the early research of Meskaoui and Trembaly (2009). Deficiencies in endogenous PA induced by DCHA further aggravated oxidative damage in white clover under water stress, as demonstrated by significantly higher carbonyl content, MDA content, and EL levels, but it could be rescued by exogenously applied Spd. Previous finding has also demonstrated that the inhibition of PA biosynthesis in pear shoots resulted in a considerable reduction in stress tolerance (Wen et al., 2011). These data indicate that PA plays a positive role in white clover tolerance to water stress, and it could be an adaptive response that increasing endogenous PA levels confer white clover a certain stress tolerance when subjected to water stress.

### PA is involved in water stress-induced cytosolic free Ca^2+^ and H_2_O_2_ production

Despite the knowledge of PA's involvement in stress tolerance, PA-regulated signal transduction pathways and molecular mechanisms associated with stress tolerance still remain unclear. It has been well-documented that the production of H_2_O_2_ or Ca^2+^ signaling molecules induce plant defense mechanisms under abiotic stress (Zhou and Guo, 2009; Gonzalez et al., 2012). A growing number of findings indicate that the interactions among PA, H_2_O_2_, and Ca^2+^ are related to plant growth and development or biotic stress such as herbivore-induced volatiles in bean (*Phaseolus lunatus*) (Ozawa et al., 2009), pollen tube growth in *Pyrus pyrifolia nakai* (Wu et al., 2010), ethylene-induced stomatal closure in *Arabidopsis thaliana* (Hou et al., 2013), and cell death in tobacco (Iannone et al., 2013). In this study, the data showed exogenous Spd could induce an instantaneous increase in H_2_O_2_ or cytosolic free Ca^2+^ in cells, and activate NADPH oxidase and *CDPK* gene expression. To a great extent, DCHA could reduce the water stress-induced H_2_O_2_ or cytosolic free Ca^2+^ accumulation, while the depressive effects of DCHA were alleviated by the application of exogenous Spd under water stress. An earlier study in *Vicia faba* showed that Put exhibited similar effects with ABA on regulation of H_2_O_2_ production as well as cytosolic free Ca^2+^ level, which resulted in stomatal closure (An et al., 2008). These findings suggest that, at least in part, PA is involved in water stress-induced production of H_2_O_2_ and Ca^2+^ signaling. Furthermore, it's shown that Ca^2+^ activated H_2_O_2_ production by increasing NADH, and accordingly, H_2_O_2_ induced Ca^2+^ release via oxidation of cysteine in human cells (Camello-Almaraz et al., 2006; Hidalgo and Donoso, 2008). According to obtained results in this study, Spd-induced H_2_O_2_ production required Ca^2+^ release, but at the same time, Spd-induced Ca^2+^ release was also essential for H_2_O_2_ production, which meant that there was a potential mutual influence between PA-induced H_2_O_2_ and Ca^2+^ signaling. Gonzalez et al. (2012) also found that cross talk between calcium message systems and H_2_O_2_ was involved in copper-induced antioxidant enzyme gene transcripts such as APX and GST. Results from the present study suggest that PA could be involved in water stress-induced H_2_O_2_ and cytosolic free Ca^2+^ production, contributing to improved stress tolerance in white clover. Previous studies have proved that ABA-induced H_2_O_2_, as signaling molecule, accumulated in stoma associated with stoma closure (Zhang et al., 2001; Bright et al., 2006). Interestingly, similar result was observed in this study. H_2_O_2_ accumulation increased rapidly in stomas of treated leaves, which may imply that Spd-induced H_2_O_2_ was associated with stoma closure under water stress, but accurate mechanisms still need to be further studied.

### Pa, H_2_O_2_,and Ca^2+^ messenger systems are involved in water stress-induced antioxidant defense and dehydrin genes expression

Antioxidant defense systems play a fundamental role in plant tolerance of oxidative damage. Under drought stress, a drought-resistant white clover cultivar exhibited significantly elevated antioxidant defenses relative to a sensitive one (Li et al., 2013a). Drought preconditioning improved drought tolerance of white clover associated with enhancing SOD activity and gene transcript level (Li et al., 2013b). Our previous studies also showed that exogenous PA improved antioxidant enzyme activities and relevant genes expression such as *SOD, GPOX, CAT*, and *APX* in white clover during drought conditions (Li et al., 2014a,b). From the results of the present study, it was obvious that the treatment with PA biosynthesis inhibitor “DCHA” effectively inhibited water stress-induced antioxidant enzyme activities and gene expression, while the effects were reversed with the exogenously applied Spd to a great extent. This indicates that water stress-induced antioxidant defense involves PA biosynthesis.

However, whether PA is involved in water stress-induced antioxidant defenses through regulating H_2_O_2_ and Ca^2+^ signaling in white clover, thereby gaining drought tolerance is not clear. The up-regulation of PA oxidation induced the release of H_2_O_2_ in tobacco, which was related to changes in antioxidant enzyme activities (Iannone et al., 2013; Guo et al., 2014). However, at least two more pathways could explain the accumulation of H_2_O_2_ in plant cells besides PA oxidation: one is NADPH oxidase, the other is cell wall-localized peroxidase. Specific H_2_O_2_-generating pathways will be activated when plants suffer from different stimuli (Neill et al., 2002; Guo et al., 2014). Apart from the inhibitor of amine oxidase “QC,” both inhibitors of NADPH oxidase “DPI” and cell wall-localized peroxidase “SHAM” inhibited Spd-induced antioxidant enzyme activities as well as gene transcript levels including *SOD, GPOX, CAT*, and *APX* in leaves of white clover, demonstrating that both cell wall-localized peroxidase and NADPH oxidase were related to Spd-induced antioxidant defense. This further supports previous studies which PA-induced antioxidant defense is related to H_2_O_2_ in plants under water stress. H_2_O_2_ generation induced by PA during early phases of stress response has a regulatory role in activate antioxidant enzymes and genes expression resulting in improvement of stress tolerance via maintaining the balance between the generation and quenching of ROS in plant cells. It has been shown that the interaction between Ca^2+^ and PA was relative to calcium-sensing receptors in cardiac tissues (Wang et al., 2003). Despite this, little is known about effects of PA on the induction of calcium sensors to regulate antioxidant defenses in plants. In this study, not only two calcium channel blockers but also a CaM antagonist and an inhibitor of CDPK effectively inhibited the Spd-induced increases in antioxidant enzyme activities as well. These changes in enzyme activities were in accordance with gene transcript levels encoding antioxidant enzymes. Thereby, obtained findings imply that both Ca^2+^ and calcium sensors could be involved in PA-induced antioxidant defense.

The abundance and gene transcript level of dehydrins are altered by water stress associated with drought tolerance in plants (Bian et al., 2002; Hu et al., 2010). Various phytohormones or physiological activators could induce the expression of dehydrins such as ABA, cytokinin, and proline, which means there are a potential interaction between dehydrins and multiple stresses signaling in plants (Wang et al., 2002; Khedr et al., 2003; Cerny et al., 2011). A drought resistant white clover cultivar maintained higher dehydrin gene expression and content than the sensitive one under drought stress (Vaseva et al., 2011). Our recent study also showed that exogenous Spm-induced dehydrins synthesis in two different white clover cultivars was responsible for improved drought tolerance (2015b). It's obvious at present that dehydrin genes *Y*_2_*SK, Y*_2_*K*, and *SK*_2_ were shown to be highly responsive to exogenous Spd. But Spd-induced enhancement of these gene transcript levels was inhibited by application of H_2_O_2_ scavenger or the inhibitors of NADPH oxidase and CDPK. It is suggested that PA-regulated H_2_O_2_ and CDPK signaling are involved in dehydrin genes expression in white clover. The result from this study highlights the function of PA on improvement of tolerance to water stress.

In summary, our results reveal that PA involvement in the regulation of H_2_O_2_ and Ca^2+^ messenger systems results in tolerance to water stress associated with antioxidant defense and dehydrins in leaves of white clover. In addition, it's worth pointing out that further investigations are needed for PA-induced stress tolerance associated with H_2_O_2_ and Ca^2+^ signaling in different metabolic pathways, plant species and stress conditions.

### Conflict of interest statement

The authors declare that the research was conducted in the absence of any commercial or financial relationships that could be construed as a potential conflict of interest.
