# Corrigendum: Polyamine regulates tolerance to water stress in leaves of white clover associated with antioxidant defense and dehydrin genes via involvement in calcium messenger system and hydrogen peroxide signaling

**DOI:** 10.3389/fphys.2016.00052

**Published:** 2016-02-15

**Authors:** Zhou Li, Yan Zhang, Dandan Peng, Xiaojuan Wang, Yan Peng, Xiaoshuang He, Xinquan Zhang, Xiao Ma, Linkai Huang, Yanhong Yan

**Affiliations:** Department of Grassland Science, College of Animal Science and Technology, Sichuan Agricultural UniversityChengdu, China

**Keywords:** antioxidant, dehydrin, gene expression, oxidative damage, polyamine, regulation, white clover (*Trifolium repens*)

In the paper titled “Polyamine regulates tolerance to water stress in leaves of white clover associated with antioxidant defense and dehydrin genes via involvement in calcium messenger system and hydrogen peroxide signaling,” there was a typing error in the section of “Introduction,” which should be corrected. In the first paragraph in the section of “Introduction,” “putrescine (Put), spermidine (Spd) and spermine (Spd)” should be corrected to “putrescine (Put), spermidine (Spd) and spermine (Spm).” No other correction is needed.

## Author contributions

All authors listed, have made substantial, direct and intellectual contribution to the work, and approved it for publication.

### Conflict of interest statement

The authors declare that the research was conducted in the absence of any commercial or financial relationships that could be construed as a potential conflict of interest.

