# Supplementary material for: Polyamine regulates tolerance to water stress in leaves of white clover associated with antioxidant defense and dehydrin genes via involvement in calcium messenger system and hydrogen peroxide signaling
Source: Front Physiol. 2015 Oct 12;6:280. doi: 10.3389/fphys.2015.00280 (PMC4600907; doi:10.3389/fphys.2015.00280)
Supplement: Supplementary file 1 [file DataSheet1.DOC]

**Figure S1** Polyethylene glycol (PEG) 6000 concentration-dependent induction on SOD (A), GPOX (B), CAT (C) and APX (D) activities in detached leaves of white clover. The detached leaves were pre-treated with distilled water for 1 h to eliminate wound stress and then exposed to 0%, 10%, 15%, 20%, 25% PEG solution for 8 h. Means of six independent samples are presented. Bars represent standard error. The same letter above columns indicates no significant difference (LSD) at *P<* 0.05.

**Figure S2** Spermidine (Spd) concentration-dependent induction on SOD (A), GPOX (B), CAT (C) and APX (D) activities in detached leaves of white clover. The detached leaves were pre-treated with distilled water for 1 h to eliminate wound stress and then exposed to 0, 15, 20, 25, 30 μM Spd solution for 8 h. Means of six independent samples are presented. Bars represent standard error. The same letter above columns indicates no significant difference (LSD) at *P<* 0.05.

**Figure S3** Time course of PEG or Spd-dependent induction on *SOD* (A), *GPOX* (B), *CAT* (C) and *APX* (D) gene relative expression levels in detached leaves of white clover. The detached leaves were pre-treated with distilled water for 1 h to eliminate wound stress and then exposed to distilled water (control) or 15% PEG or 20 μM Spd solution for 8 h. Means of four independent samples are presented. Bars represent standard error. The same letter above columns indicates no significant difference (LSD) at *P<* 0.05.
